# Supplementary material for: Investigation of Alogliptin-Loaded In Situ Gel Implants by 23 Factorial Design with Glycemic Assessment in Rats
Source: Pharmaceutics. 2022 Sep 5;14(9):1867. doi: 10.3390/pharmaceutics14091867 (PMC9501034; doi:10.3390/pharmaceutics14091867)
Supplement: Supplementary file 1 [file pharmaceutics-14-01867-s001.zip › pharmaceutics-1848816-supplementary.pdf]

Supplementary data

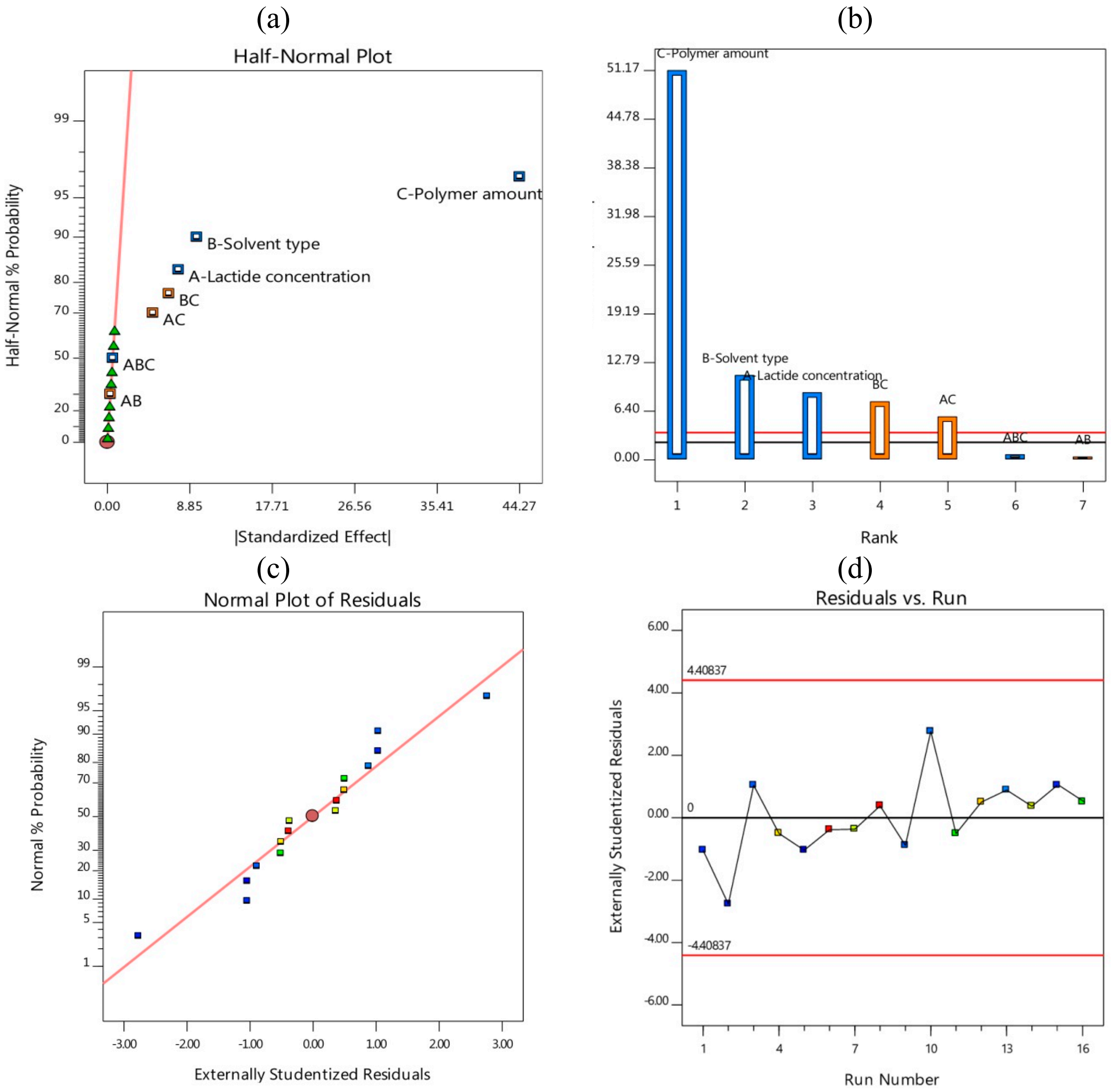

Figure S1: Determination of order of significant factors on Y_1 response including (a) half-normal plot (b) Pareto chart, and analysis of model diagnostic plots including (c) normal plot of residuals (d) residuals versus runs plot.

(a) (b)

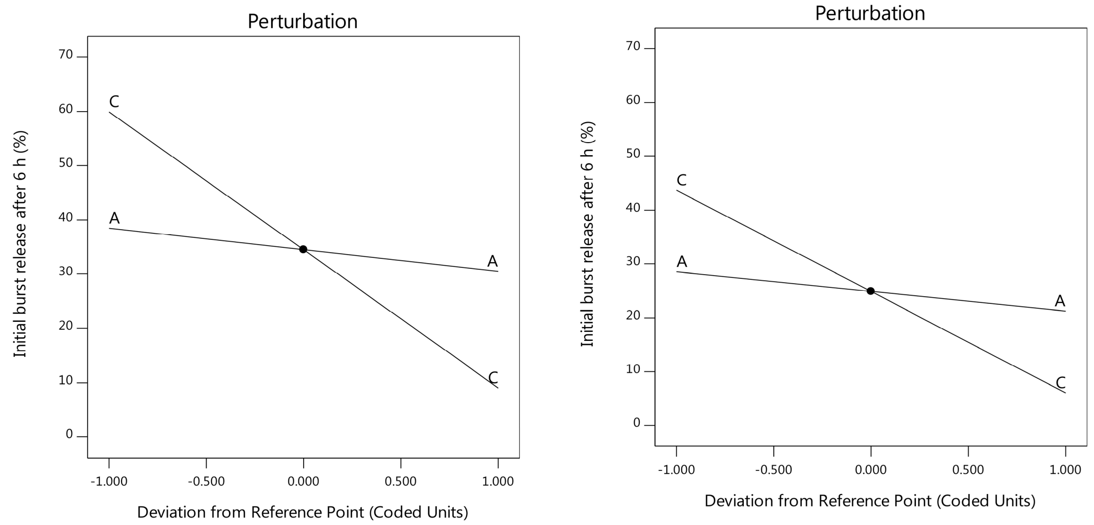

Figure S2: Perturbation plot of influence of independent factors on Y_1 response (a) effects of A and C using DMSO (b) effects of A and C using NMP.

In the perturbation plot, a resulted steep slope or curvature could express the significant sensitivity of the response to the factor, while a relatively flat line could indicate the non-significance of the studied factor on the measured response **[59]**. The perturbation plots by using DMSO solvent (Figure S2 (a) supplementary data) or NMP solvent (Figure S2 (b) supplementary data) had showed the incredible effect of increasing the PLGA amount on decreasing the initial alogliptin burst release after 6 h in comparison to the low significant effect of increasing lactide concentration of PLGA on the tested burst release. This could be deduced from the steeper slope of factor C than that of factor A. Therefore, increasing the PLGA amount could be more required to decrease the initial burst release than other independent variables.

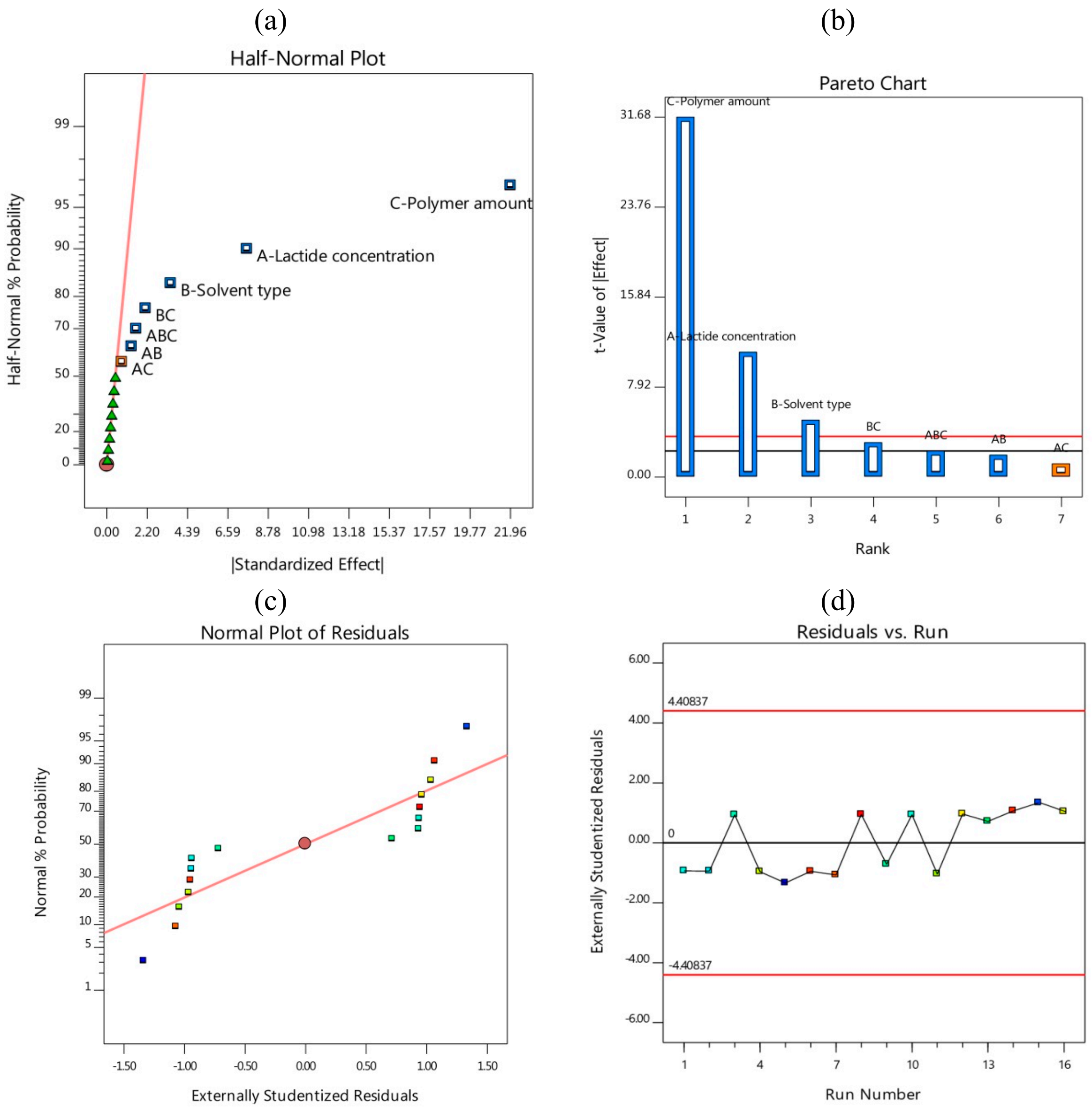

Figure S3: Determination of order of significant factors on Y_2 response including (a) half-normal plot (b) Pareto chart, and analysis of model diagnostic plots including (c) normal plot of residuals (d) residuals versus runs plot.

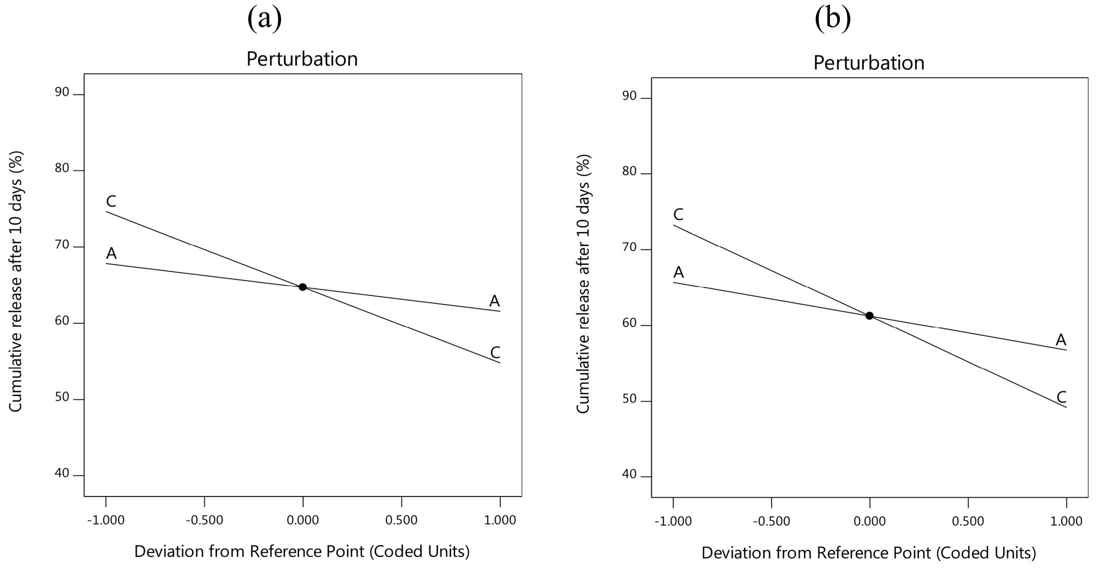

Figure S4: Perturbation plot of influence of independent factors on Y_2 response (a) effects of A and C using DMSO (b) effects of A and C using NMP.

The perturbation plots were carried out where a steep slope of factor could indicate the higher sensitivity of the cumulative release of drug to such factor. By using DMSO solvent (Figure S4 (a) supplementary data) or NMP solvent (Figure S4 (b) supplementary data), the outstanding effect of increment of PLGA amount on decreasing the cumulative release of alogliptin after 10 days was observed in comparison to the lower significant effect of increasing factor A on the tested Y_2 response. Hence, the alogliptin release from ISGI formulations was highly sensitive to alterations of solvent to PLGA ratios.

References

59. Kumar, L.; Reddy, M.S.; Managuli, R.S.; Pai K.G. Full Factorial Design for Optimization, Development and Validation of HPLC Method to Determine Valsartan in Nanoparticles. *Saudi Pharm. J.* **2015**, *23*, 549–555. https://doi.org/10.1016/j.jsps.2015.02.001.
